# Prevalence of insulin resistance in obese adolescence

**DOI:** 10.1186/1687-9856-2013-S1-P102

**Published:** 2013-10-03

**Authors:** Ardita Puspitadewi, Rini Sekartini, Aman B Pulungan

**Affiliations:** 1 Department of Child Health, Faculty of Medicine University of Indonesia, Jakarta

## 

Childhood obesity is a global health problem with several metabolic and cardiovascular complications. The prevalence of childhood and adolescent obesity is differed in each country with several affecting factors. Insulin resistance as one common feature of obesity is known to have a link with the risk of diabetes type 2 and cardiovascular complication. Insulin resistance is also a basic mechanism of metabolic syndrome. Several mechanisms proposed the link between obesity, insulin resistance, and metabolic syndrome. This study aimed to look at the characteristics of obese adolescence with metabolic syndrome and to look for the prevalence of insulin resistance among them with several risk factors that may affect.

This was a cross-sectional study on 92 obese adolescent, aged between 12 and 15 years old, in Central Jakarta, Indonesia. All adolescent underwent blood sample tests, including fasting blood glucose, lipid profile, and fasting blood insulin. Insulin resistance was defined as a HOMA-IR index of 3.8 or more. The diagnostic of metabolic syndrome was defined according to International Diabetes Federation (IDF) criteria.

Insulin resistance was found in 38% of the adolescent, with a higher rate among females (57.2%) than males (42.8%) (p = 0.673). Most of them had acanthosis nigricans as the marker of insulin resistance (71.4%) (p = 0.959). Among them, 82.8% had a family history of metabolic syndrome (p = 0.646). The prevalence of metabolic syndrome among obese adolescent was 19.6% with female predominant. The prevalence of each of the component was 48.9% for high blood pressure, 78,3% for abdominal obesity, 8.7% for impaired fasting glucose level, 22.8% for low levels of high-density lipoprotein cholesterol, and 21.7% for high triglyceride level. There was a strong correlation between impaired fasting glucose level and insulin resistance (p = 0.04), with the risk of 5.7 times to get insulin resistance.

Insulin resistance has a prevalence of 38% in obese adolescent population in this study. Insulin resistance has a significant association with impaired fasting glucose level.

